# Supplementary material for: Segregation of endoderm and mesoderm germ layer identities in the diploblast Nematostella vectensis
Source: Nat Commun. 2025 Aug 27;16:7979. doi: 10.1038/s41467-025-63287-4 (PMC12381260; doi:10.1038/s41467-025-63287-4)
Supplement: Supplementary file 1 — Supplementary information [file 41467_2025_63287_MOESM1_ESM.pdf]

SUPPLEMENTARY INFORMATION

Segregation of endoderm and mesoderm germ layer identities in the diploblast *Nematostella vectensis*

Emmanuel Haillot, Tatiana Lebedeva, Julia Steger, Grigory Genikhovich, Juan D. Montenegro, Alison G. Cole, Ulrich Technau

Contents

Supplementary Figures 1-6

Supplementary Table 1

Supplementary Figures

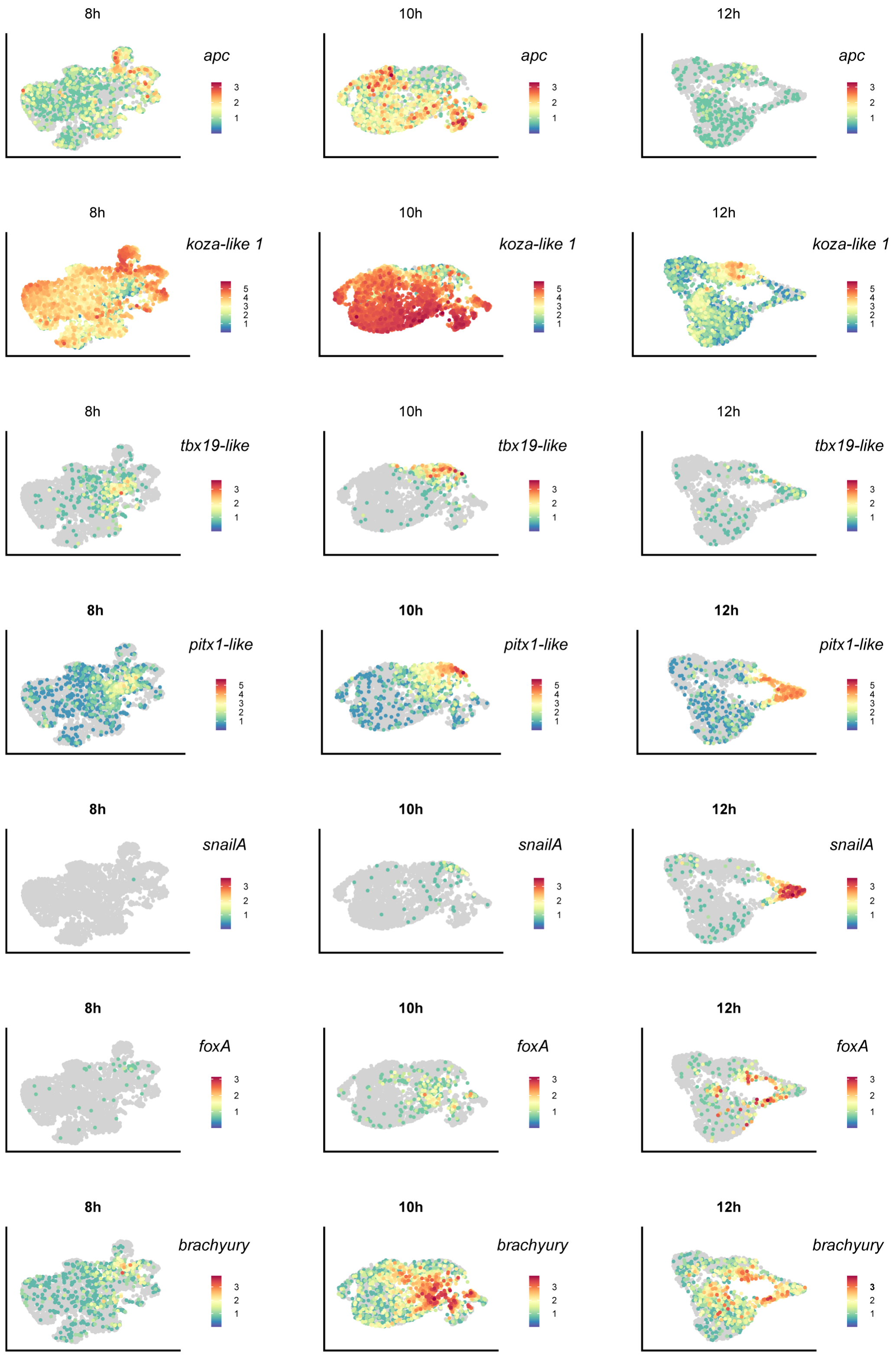

Supplementary Fig. 1 UMAP projections of single RNAseq datasets at 8, 10 and 12hpf. Visualization of the cells expressing ectodermal genes (*apc*, *koza-like 1)*, mesodermal genes (*tbx19-like*, *pitx1-like, snailA)* and endodermal genes (*foxA*, *brachyury)* at different time points during early development in *Nematostella*.

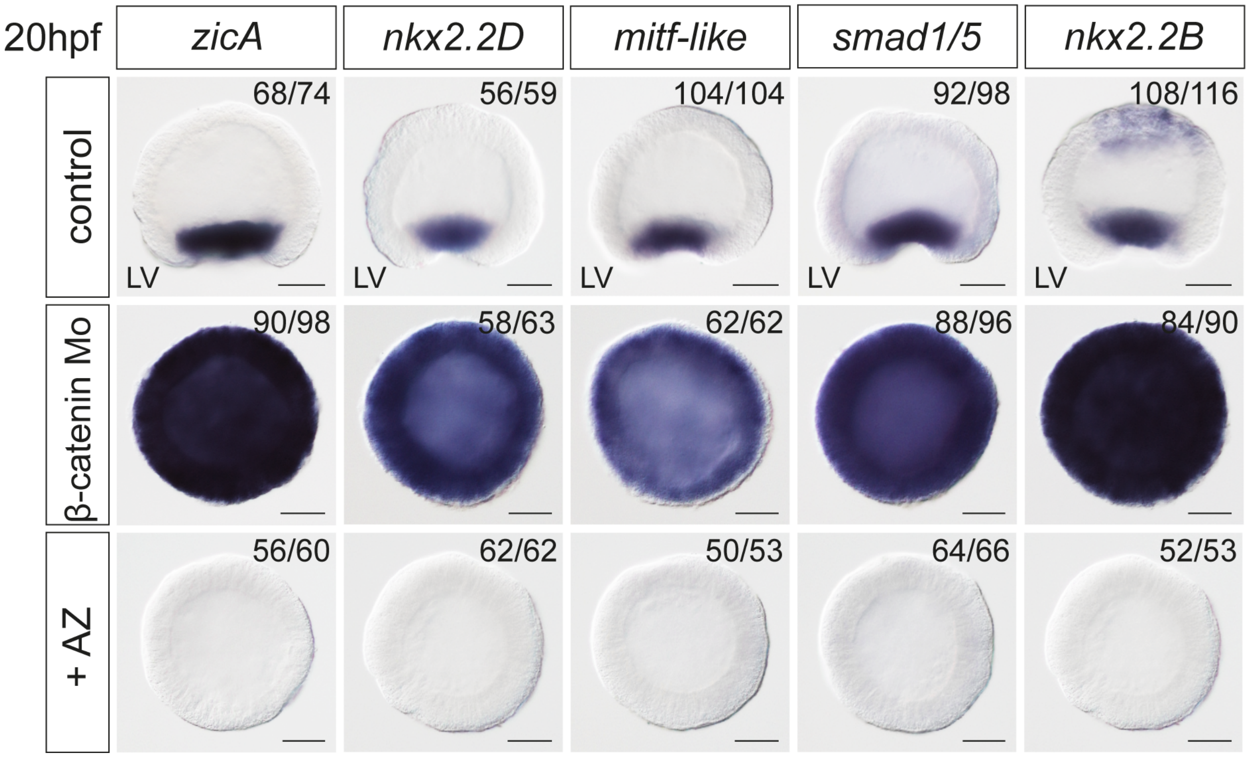

Supplementary Fig. 2 β-catenin signaling prevents mesodermal marker expression at 20 hpf. Expression of mesodermal genes *zicA*, *nkx2.2D*, *mitf-like*, *smad1/5*, *nkx2.2B* in AZ treated embryos or β-catenin morpholino injected embryos at 20hpf. Scale bar 50μm. LV; lateral view. All treatments were replicated three times with similar results.

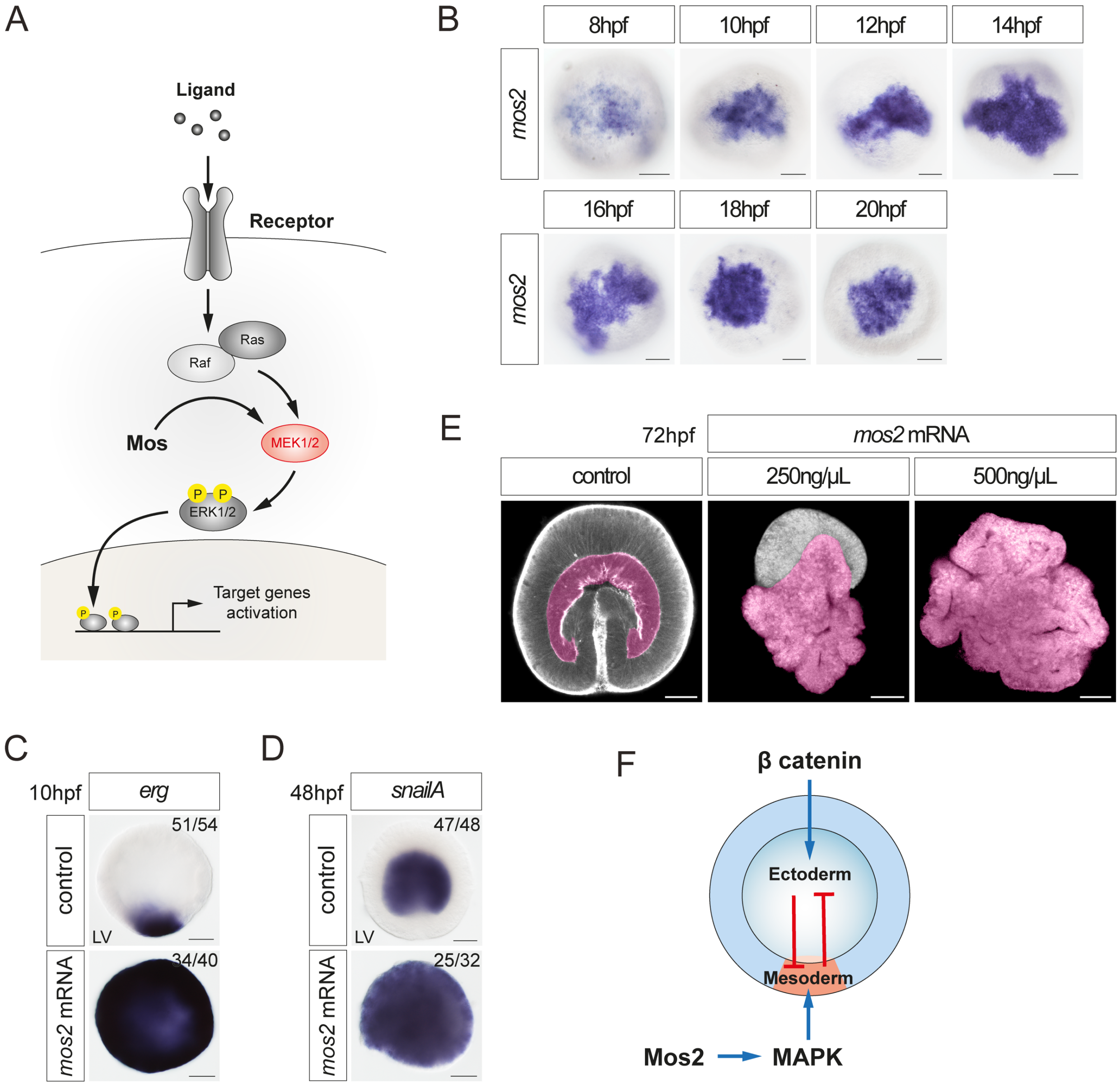

Supplementary Fig. 3 The kinase Mos2 promotes the mesodermal fate. (A) Scheme of the RTK/MAPK signaling pathway showing the role of the serine/threonine kinases Mos in the MAPK signaling pathway. (B) Spatial and temporal expression profile of *mos2* revealed by in situ hybridization. All pictures show oral view. Scale bar 50µm. (C) Overexpression of *mos2* induces ectopic expression of the mesodermal gene *erg* at 10hpf. Scale bar 50µm. LV; lateral view. (D) The effect of *mos2* overexpression can still be observed at 48hpf with the ectopic expression of the mesodermal gene *snailA* in whole embryo. Scale bar 50µm. LV; lateral view. (E) Morphological analysis on *mos2* mRNA injected embryos stained with phalloidin (gray). Mesodermal cells are

highlighted in magenta. Scale bar 50µm. (F) Schematic summary of the potential role of Mos2 in the activation of the MAPK signaling and of the mutual antagonism between β-catenin and MAPK signaling. All experiments were replicated three times with similar results.

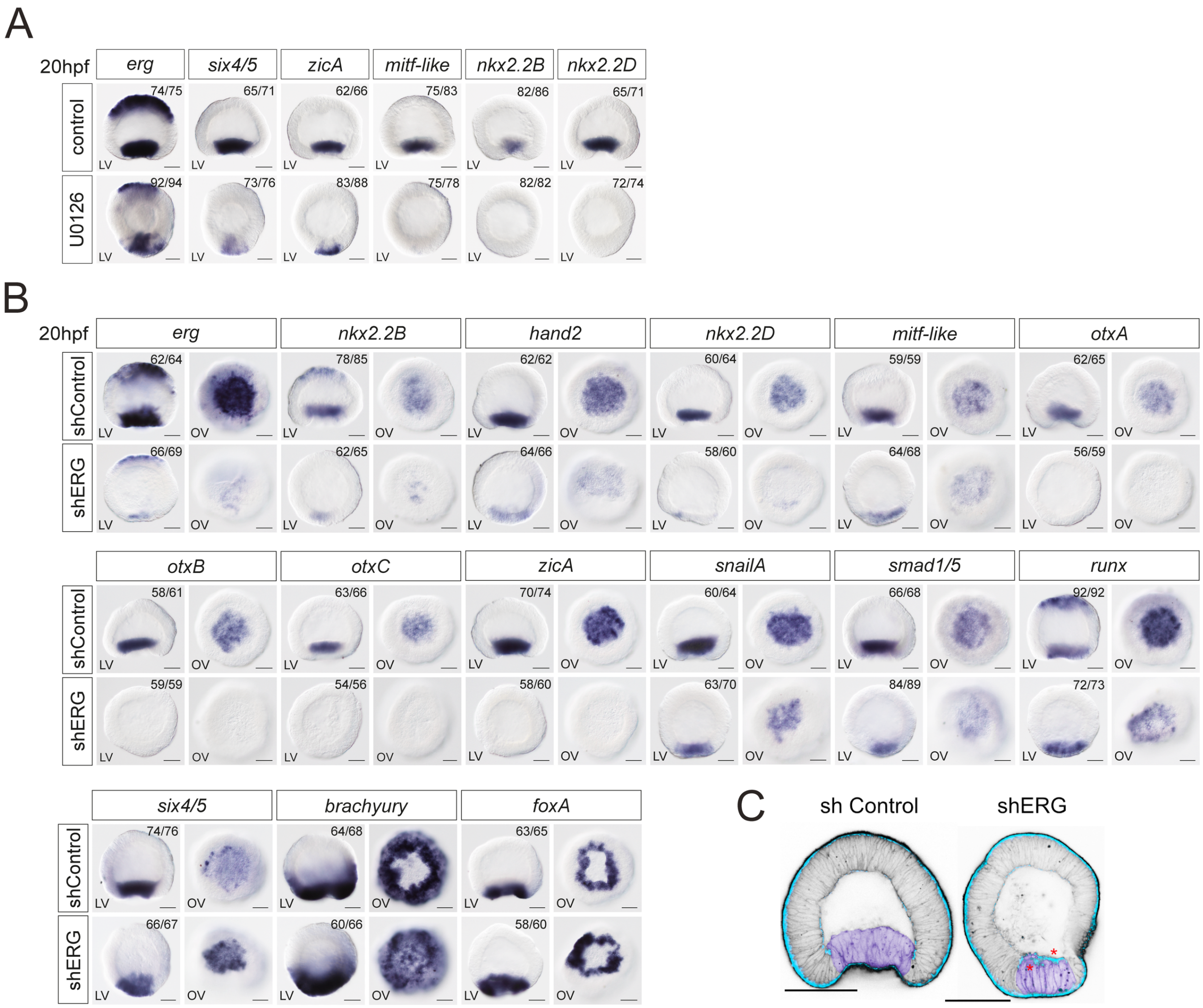

Supplementary Fig. 4 ERG is essential for mesoderm specification and morphogenesis. (A) Mesodermal gene expression analysis by in situ hybridization on 20hpf U0126 treated and control embryos. Scale bar 50µm. LV; lateral view. (B) Mesodermal (*erg*, *nkx2.2B*, *hand2*, *nkx2.2D*, *mitf-like*, *otxA*, *otxB*, *otxC*, *zicA, snailA, smad1/5, runx*, *six4/5*) and endodermal (*brachyury*, *foxA*) gene expression analysis by in situ hybridization on 20hpf shERG injected embryos. Scale bar 50µm. LV; lateral view, OV; oral view. (C) The effect of the *erg* knockdown on cdh3 expression (blue) and on gastrulation movements phenocopies the effect of the MAPK signaling inhibition by U0126. F-actin (black) staining is used to show the cell outlines. Scale bar 100µm. All treatments were replicated three times with similar results.

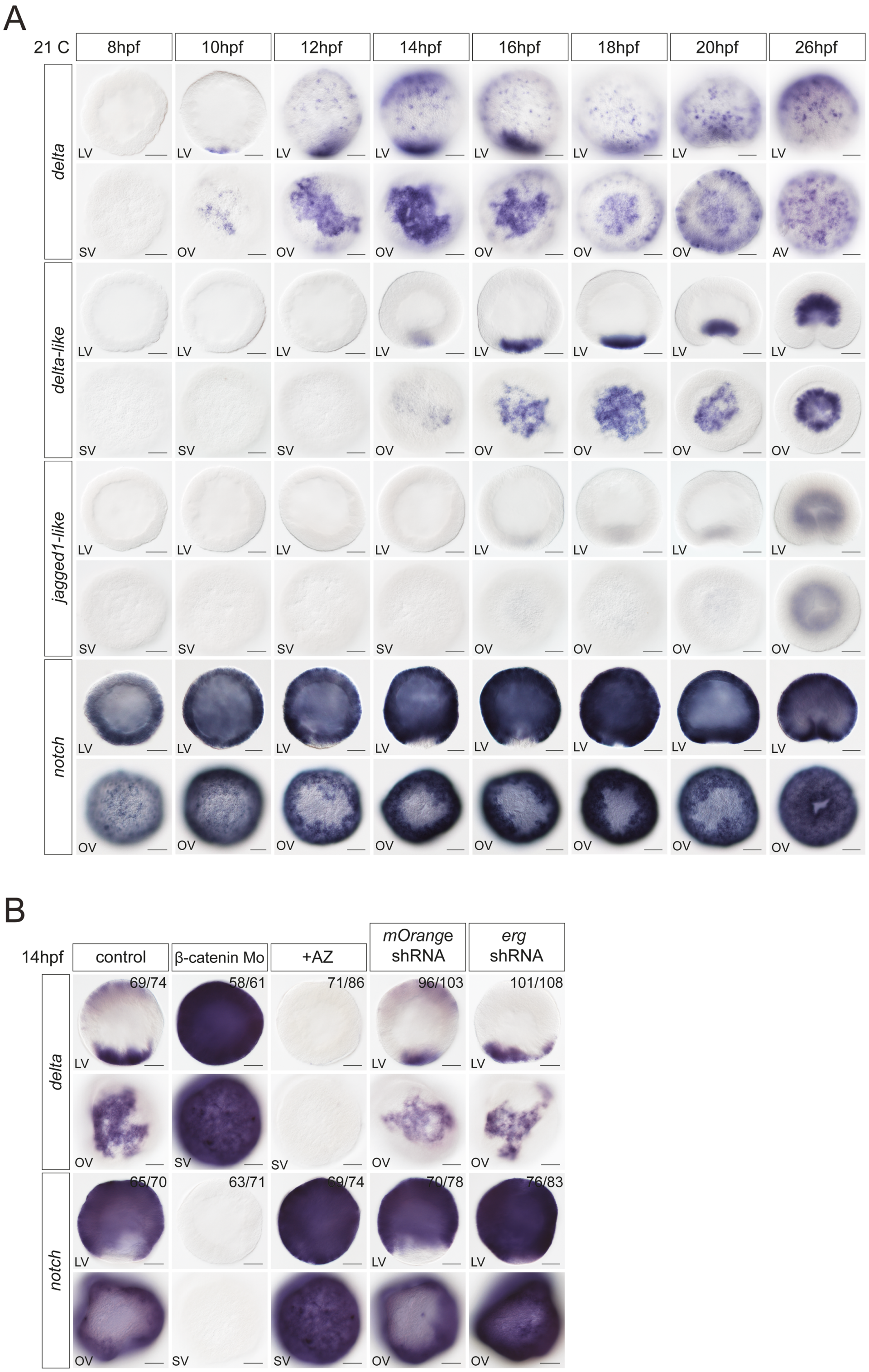

Supplementary Fig. 5 Regulation of ligands and receptor of the Notch pathway. (A) Expression analysis of genes coding for ligands (*delta, delta-like, jagged1-like*) and receptor (*notch*) of the Notch pathway from 8hpf to 26hpf by in situ hybridization. Scale bar 50μm. LV;

lateral view, OV; oral view. (B) In situ hybridization at 14hpf in AZ treated embryos, β-catenin morpholino or shRNA against *erg* injected embryos to visualize mesodermal (*delta*) and ectodermal (*notch*) genes expression. Scale bar 50μm. LV; lateral view, OV; oral view, SV; surface view. All treatments were replicated three times with similar results.

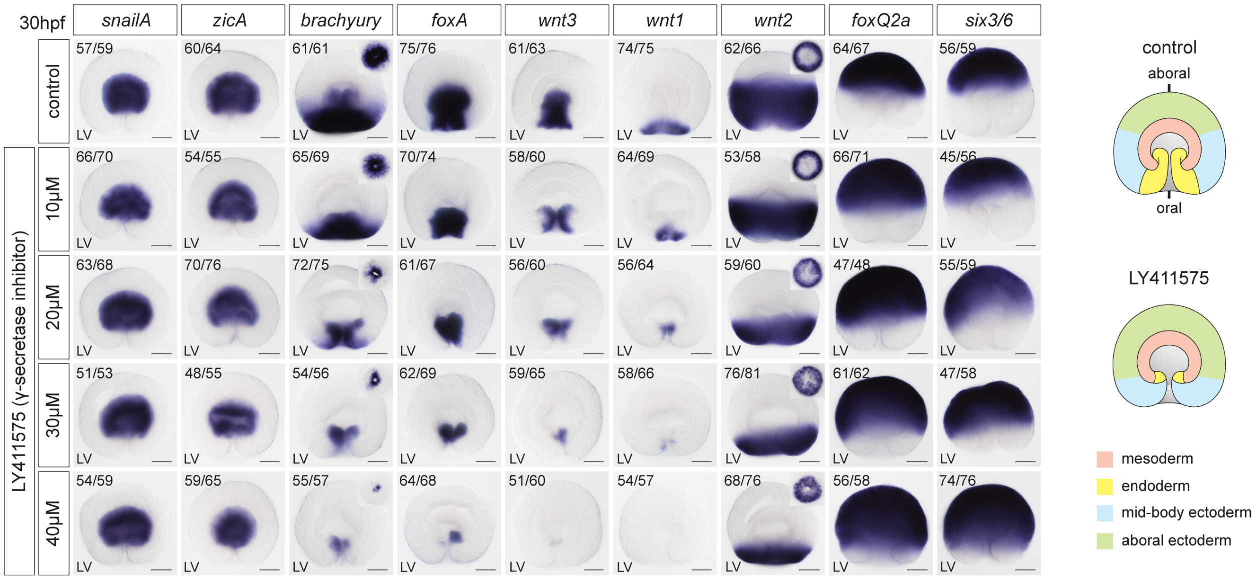

Supplementary Fig. 6 Notch signaling is essential for endodermal gene expression. Mesodermal (*snailA, zicA*), endodermal (*brachyury, foxA, wnt1, wnt3, wnt1*) and ectodermal (*wnt2, foxQ2a, six3/6*) gene expression analysis at 30hpf on embryos treated with LY411575 using in situ hybridization; Expression of endodermal genes is downregulated after inhibition of the Notch signaling. Scale bar 50μm. The inlet added for *brachyury* and *wnt2* is an oral view LV; lateral view, OV; oral view. The experiment was replicated three times with similar results

Supplementary Table 1 Oligonucleotide primers used in this study.

Nematostella vectensis genes	NVE	NV2	Forward Primers	Reverse PRIMERS
tbx19-like	NVE695	NV2.15833	TGCAATGATGGCTGACACTG	GTTTCCTCTTCCGTAACGCC
gsc2-like	NVE11074	NV2.2151	CATGAACAATACAACCTGCG	CTGTTATGCGATGATTCAGC
duxABC 1	NVE13066	missing	AAAATAATCCAGTACCGCCC	CCTTGTGCCTAAGTTATAACG
duxABC 2	NVE13067	NV2.1307	GAAACGCACCAACCCTTA	CGTAATAACTTAGGCACAAGG
koza-like 1	NVE2478	NV2.234	CCAACAAGAACACAAACACA	GCCTCTACCCATGATGTTTT
koza-like 2	NVE2480	NV2.234	CCAACAAGAACACAAACACA	TAAACTGATTCTGTGCGTCT
bHLH TF-like	NVE19743	NV2.10891	CGAGAAAGATCAGTACCACT	CAATCGAAGCTTTTCTGTCC
apc	NVE16658	NV2.15303	ATTCTCAATCGACGCCATC	GTTGTTTCCGCTCTGCTTTC
pitx1-like	NVE15108	NV2.9419	GAAACGCACCAACCCTTA	CGTAATAACTTAGGCACAAGG
fgfa1	NVE11443	NV2.8484	TGCACTTTTGTCCAGAGAG	AGCTTAAACGCCCTTCTAAA
erg	NVE25536	NV2.22508	AATGTTGTTCCCGATGCGT	CCCACCTTCTTGCTACCTCA
nkx2.2B	NVE11289	NV2.11262	GTGAAGCCATGAGTCAAGTA	TATGAACCGGTACTCTGTCT
zicA	NVE25610	NV2.22580	ACGTGGTTTCACCAGCTA	GCCAACCATTGATTCTTGCT
snailA	NVE13986	NV2.472	ATGCCCCGCTCGTTTCTAG	CTATCCTTGTGACGGGCA
mitf-like	NVE15793	NV2.3755	GAAAGGGTAAGGTTTGCAGT	GATCAGTGCCAGATTGTACT
runx	NVE11457	NV2.8464	GGAGAAATAAGCGGAAGAGA	TCTAATACGGTCTCCACACA
six4/5	NVE17554	NV2.10728	CGGACTTGATTCACTAAGGGC	AGTGTAGGTACGACTCCTCAGG
smad1/5	NVE5479	NV2.2977	CCGAAAAGAGCGATACTAGT	AGTTAGTGAAAAGCGGCTAG
hand2	NVE24170	NV2.2672	CTGACTTGATCAGCGCTGCTGAAT	GGGTCGATTGTTAACATTTATTAACTTCT
zc3h12-like	NVE1859	NV2.5965	GAAATAGCCGTGCAAAATGA	TGAATGCCACTTAACTCACA
nkx2.2D	NVE10557	NV2.11134	GAGCCATGACTTCGTTCTC	TCTTAACCCTACCAAGTCCA
otxB	NVE7116	NV2.12795	TCAAGTAGGCAAGCAAGAC	GCTTTTCATTTAAATCCACGG
isx-like 2	NVE20118	NV2.17634	TCATCATACGTTTGTCTCTCC	GCTTCTGGGTCTTCAAGAG
hmx2	NVE1454	NV2.25557	CTTTCTCCACGCCACATAC	AGATAGTGAAGAGTCCGTGA
otxC	NVE7117	NV2.12793	ATCTCCGAACTTCGACTACTA	TAGGTATAAACTACTGCTGTCGTA
brachyury	NVE3568	NV2.10624	ATGCACTCGGACGAGAAGAAAC	TTAAGCTTGCGGTATGGTGTTCC
foxA	NVE20630	NV2.11441	ATGATGGAGCACACGGG	CGAAAGATTTGATATACCACAACTC
wnt1	NVE12960	NV2.12225	ATGCAACGATTCAGCGCAGCGATC	TTATAAGCAGTTACTAATGATCC
wnt3	NVE17595	NV2.12463	ATGAGAGTAATTATCACTGCGATTG	TTATTTACAAGTGTAGATGTTAACCACTT
otxA	NVE7115	NV2.12799	AATAGTACACCCACGACATC	CTGTGCTTCTGTGAAATTCC
delta	NVE17145	NV2.20616	CAATCGGTACACCTGCTC	CAAGTTCCATCACCAAATGC
delta-like	NV5234	NV2.4253	GTCTTTTGGAGTCGCTGTGG	GCTCCCGTTAGAATCACACG
jagged1-like	NVE10960/NVE18138	NV2.9243	AGTTGGAATACTGGCTTGTT	CTGATTGTTGTGTTGTGCTT
notch	NVE9611	NV2.24622	AGCTGAGAAGATTTGGTTCATTG	TTGACCAGTCTGATAATAACTCCA
foxQ2a	NVE14268	NV2.13108	TAGCAGTCAGTAGTCGCACAG	ACATAGACCCTGTCCTGGC
six3/6	NVE12346	NV2.12726	ATGTTCGCACCGTTACCAGCG	TCAGGGCCGCTGCTCGGAGAG
